# Evaluation of a “one-stop shop” for integrated harm reduction and primary care for people who inject drugs

**DOI:** 10.1371/journal.pone.0337528

**Published:** 2025-11-21

**Authors:** Nadeen Ibrahim, Shaifer Jones, Katherine Rich, Lisandra Alvarez, Carolina Price, Natalie Kil, Frederick L. Altice, Jaimie P. Meyer

**Affiliations:** 1 NIDA Summer Internship Program, Bethesda, Maryland, United States of America; 2 Yale School of Medicine, New Haven, Connecticut, United States of America; 3 Harvard Medical School, Boston, Massachusetts, United States of America; 4 Department of Medicine, Section of Infectious Diseases, Yale School of Medicine, New Haven, Connecticut, United States of America; 5 Epidemiology of Microbial Diseases, Yale School of Public Health, New Haven, Connecticut, United States of America; 6 Chronic Disease Epidemiology, Yale School of Public Health, New Haven, Connecticut, United States of America; Centers for Disease Control and Prevention, UNITED STATES OF AMERICA

## Abstract

**Background:**

People who inject drugs (PWID) experience high risk for HIV and HCV infection, which can be mitigated by harm reduction strategies, including syringe service programs (SSP). Understanding individuals’ patterns of substance use and SSP utilization is important for optimizing harm reduction strategies and disease prevention for PWID.

**Methods:**

We evaluated demographic characteristics and service utilization from the New Haven Syringe Services Program (NHSSP), a low-threshold service delivery site in New Haven, Connecticut that provides fully integrated harm reduction and primary healthcare services to PWID. Site-specific data were extracted from the e2ctprevention database, managed by the Connecticut Department of Public Health, and EvaluationWeb from January 2017 to October 2023. We conducted a descriptive analysis of basic demographic and social characteristics of SSP clients, transaction characteristics, and service utilization. Statistical analyses were conducted using STATA v 16.1 and IBM SPSS Statistics (v 29.0.2.0).

**Results:**

Among 1,189 unique individuals utilizing SSP during the observation period, most (65.2%) identified as men and white (73.3%), consistent with SSP clients regionally and nationally. The mean age of clients was 41 years (SD = 9.8); approximately half of participants were unstably housed and 80% were unemployed at intake. From June 2020 to October 2023, there were 7,238 transactions, which increased throughout the COVID-19 pandemic period. During this period, the program dispensed 1,860,621 syringes, in addition to other materials, including overdose education and naloxone distribution (OEND), and provided patient education on safer injecting techniques and wound care.

**Conclusion:**

In this first comprehensive analysis of a large SSP since its inception and through the COVID-19 pandemic, we described important client characteristics and utilization of an array of syringe services from an integrated SSP. Findings suggest the SSP attracts a high volume of clients, provides on-demand services, and reaches a wide range of clients. Future research is needed to evaluate the impact of the program’s home-delivery service and increased outreach efforts. Despite limitations, the program’s success demonstrates the SSP can serve as a model for other harm reduction programs nationally.

## Introduction

People who inject drugs (PWID) are at greater risk for health and social problems and experience a higher level of adverse health outcomes than the general population, both related to their substance use and because they experience more substantial barriers to accessing care [1–4]. The sharing and use of non-sterile injecting equipment increases individuals’ exposure to HIV, hepatitis C virus (HCV), other bloodborne pathogens [2,5], and skin and soft tissue infections (SSTIs), including from drug-resistant pathogens like MRSA [3,6]. Longer-term and more severe health complications of injecting are common, including HIV, cirrhosis from HCV, endocarditis, and septic thrombophlebitis [7,1]. In 2022, PWID accounted for about 1 in 14 new HIV diagnoses in the United States, and for every 100 PWID with HIV, 9 did not know their HIV status [8]. The greater burden of disease experienced by PWID is compounded by the fact that PWID are less likely to engage in primary care than the general population because of difficulty navigating healthcare systems, concerns about cost, un- or under-insurance, competing priorities (such as meeting needs related to housing, food, and addiction), and stigmatization by healthcare providers and systems [9–13]. Consequently, PWID utilize the emergency department and are hospitalized more often than the general population [14]. The COVID-19 pandemic only further exacerbated healthcare disparities for PWID, when access to care was limited. Amidst the opioid overdose epidemic in the United States, there is critical need for expanded access to integrated harm reduction and healthcare services.

Invigorated and new interventions are necessary to increase access to syringe service programs (SSPs, also known as syringe or needle exchanges), evidence-based treatment for substance use disorders, and integrated healthcare and harm reduction services for PWID [5,15,16]. SSPs are evidence-based ways to reduce HIV transmission [17] and are recommended by international [18] and domestic authorities [19].

Given the complex medical and psychiatric comorbidity of PWID, a patient-centered “one-stop shop” is one that directly provides harm reduction services, HIV and HCV testing and linkage to care, acute and continuous primary care, and links people to treatment for substance use disorders and other healthcare services (or provides it onsite) [3]. Model success depends on low-threshold services [20] that are flexible, available without an appointment (“drop-in”), free-of-charge, anonymous, and non-judgmental. ‘The Harm Minimization Clinic’ in Sydney, Australia [9], for example, provides primary healthcare through an SSP, and variations of this model have been developed in the United States [21]. The North American Syringe Exchange Network (NASEN) tracked healthcare services before and after the COVID-19 pandemic [22], but to our knowledge, integrated programs have rarely been systematically evaluated in terms of individual or aggregate health outcomes or service utilization. Understanding this paradigm of care at the granular level is necessary for optimizing initiatives to expand access to care and to prevent new HIV and HCV outbreaks among PWID. This paper describes a low-threshold care site in New Haven, Connecticut that provides integrated harm reduction and primary healthcare services to PWID and offers insights into successes and ongoing challenges to consider in the formation of similar programs.

## Methods

### Setting and program description

New Haven is a middle-sized city of 139,000 people with high rates of substance use, poverty, unemployment, HIV and overdose. In 2019, New Haven County had the highest rate of PWH compared to all other counties in the state [23] where 25% of PWH were PWID. In surrounding non-urban areas, HCV is highly prevalent among PWID, with one study reporting that more than 40% of PWID screened were HCV antibody positive [24]. Between 2017 and 2023, New Haven had 2,661 drug overdose deaths, comprising 30% of drug overdose deaths in Connecticut [25].

Yale Clinical and Community Research has operated a mobile medical clinic (MMC), known as the Community Health Care Van, since 1993 to provide free and accessible primary healthcare and HIV prevention services [26] in four underserved neighborhoods in New Haven. The program is funded through several mechanisms. For individuals who are insured, care is billed to public and private insurance companies; all copays and co-insurance are waived. For individuals without insurance, care is provided without reimbursement. The SSP component of the program is funded through a Connecticut Department of Public Health (CTDPH) Syringe Service grant. Injection equipment, overdose education and naloxone distribution (OEND), and fentanyl test strips are provided by the CTDPH and have previously been supported by service grants, including from the DHHS Substance Abuse and Mental Health Services Administration (SAMHSA), along with several private foundation grants.

### Description of locations

Harm reduction services, including syringe exchange, condom and naloxone provision, wound care and safe injection technique education are provided through three sites: 1) the MMC; 2) a brick-and-mortar clinical office; and 3) a minivan. A bilingual and bicultural (English/Spanish) care team currently includes a full-time phlebotomist/case manager, a full-time outreach worker, one full-time and one part-time nurse practitioner, and a part-time psychiatric nurse practitioner, alongside site interns and volunteers.

#### Community health care van.

**Fig 1:**
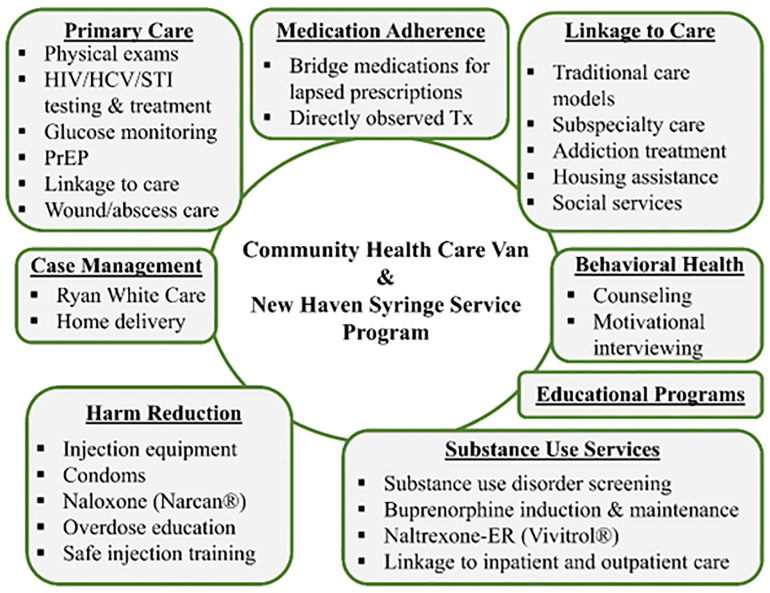
Services provided by the community health care Van-New Haven Syringe service program.

A 40-foot MMC travels to multiple locations within the City of New Haven, Monday-Friday 9am to 12 pm. The MMC has an exam room, an intake/counseling room, a waiting area, and a private area for harm reduction services. The MMC routinely screens for HIV, HCV, sexually transmitted infections, and tuberculosis (TB); monitors diabetes and hypertension; and provides acute and continuous primary care and HIV case management. Expanded services include integrated treatment for substance use disorders, directly observed HIV and TB treatment, screening and treatment for psychiatric disorders, onsite buprenorphine, extended-release naltrexone (XR-NTX), HIV pre-exposure prophylaxis (PrEP), and drug checking services using FTIR spectroscopy. The full scope of available services is shown in Fig 1. SSP services on the MMC are designed to: (1) create an integrated, low-threshold, non-judgmental model of harm reduction and healthcare services; (2) increase access to and convenience of harm reduction services; and (3) facilitate use of HIV/HCV prevention and healthcare services, as well as linkage to addiction treatment among PWID. MMC staff document clinical services, order tests and receive results through the electronic health record (EHR) of Yale-New Haven Health system. SSP utilization is not documented in the EHR to protect anonymity.

#### Fixed clinical site.

The “storefront” has two exam rooms, four counseling rooms, and a private area for SSP. Same-day buprenorphine and XR-NTX initiation are available; behavioral health practitioners are onsite and available by appointment. Clinic and SSP services are available from 8am to 4 pm daily.

#### Minivan.

The minivan provides home-delivery harm reduction services, outreach services, and linkage to substance use disorder treatment. The van travels throughout the greater New Haven area and can be contacted via phone or text. The minivan is operated by a case manager who is bilingual in Spanish and English.

#### Integration of syringe services into clinical care.

Syringes are provided as a needs-based exchange without the requirement of a 1-for-1 exchange. Clients are encouraged to return their used syringes, which encourages safe collection and disposal. Secondary exchange, meaning collection from or distribution to others, is allowed and neither encouraged nor discouraged. Behavioral healthcare is provided without charge to the patient (i.e., no copay or co-insurance) by appointment at the clinical office site. Harm reduction education is provided to individuals using various techniques, including demonstrating vein care and injection techniques and providing extensive overdose prevention counseling and naloxone. Clients are offered point-of-care rapid HIV and HCV testing at each visit.

### Measures

Site-specific data from January 2017 to December 2023 was extracted from the e2ctprevention database maintained by the CTDPH. Site-specific data from May 2019 to November 2023 on completed HIV testing and referral services was extracted from EvaluationWeb, a secure, web-based data collection and reporting system used primarily by organizations funded by the Centers for Disease Control and Prevention (CDC). Because all data is de-identified and available in a public database, the need for consent was waived. The Yale Human Investigations Committee (IRB) approved the study and deemed it exempt from further oversight.

#### Demographic and social characteristics.

During a client’s initial visit to the SSP, a ‘Client Intake Form’ records demographic information (e.g., sex at birth, gender identity, race, ethnicity, year of birth, language, zip code), social determinants of health and HIV risk (employment, housing status, medical insurance, education, and HIV risk behavior), brief substance use history, HIV status, and history of HCV. All information is self-reported, and clients are provided with a unique, anonymous code as an identifier.

#### Transaction characteristics.

At each visit, service utilization or “transaction” information is collected, which includes: the number of syringes dispensed and returned, the number of external (male) and internal (female) condoms (and other supplies) provided, if the client accepted training for safe injecting or overdose prevention, if the client ever experienced or witnessed an overdose, if a naloxone kit or prescription was provided, if a referral to substance use treatment was given, and if a referral to healthcare services was given. All transaction characteristics were analyzed in aggregate because they could not be linked back to individual clients. Transaction data was only available for encounters that occurred between June 11, 2020, and October 18, 2023.

#### Testing and evaluation services.

The EvaluationWeb HIV Testing form includes data on the site, basic client characteristics (e.g., age), type and result of HIV test, results of additional tests for co-infections (syphilis, gonorrhea, chlamydia, and HCV), and delivery of various essential support services. Testing and service data are not linked to a unique client ID so can only be assessed in aggregate.

### Statistical analysis

We conducted a descriptive analysis of basic demographic and social characteristics of SSP clients. We then conducted a descriptive analysis of transaction characteristics, service utilization and testing data, graphing the mean number of transactions per month over time. Because transaction data was available for June 11, 2020 to October 18, 2023 we were able to assess general trends in transaction volume over time and through the peak of the COVID-19 pandemic, which is important for understanding persistent delivery of essential services through the pandemic period, when many other community-based services were shut down. We also examined trends in naloxone provision as a proxy for overdose prevention services. Statistical analyses were conducted using STATA (v 16.1) and IBM SPSS Statistics (v 29.0.2.0).

## Results

### Participant characteristics

As shown in Table 1, among 1,189 individuals accessing SSP during the entire observation period (January 1, 2017- October 31, 2023), most (65.2%) identified as men and white (73.3%), consistent with SSP clients regionally and nationally. Approximately one-quarter of individuals identified as Hispanic and 11.3% identified as Black. The mean age of clients was 41 years old (SD = 9.8).

**Table 1:** Demographic and social characteristics at intake (N = 1189).

	No.	% of Total	% of Known
**Mean age, y (SD)**	41.3 (9.8)		
**Gender**			
Man	775	65.2	69.2
Woman	343	28.8	30.6
Transgender	2	0.2	0.2
Unknown/not reported	69	5.8	
**Sex at Birth**			
Male	491	41.3	68.7
Female	224	18.8	31.3
Unknown/not reported	474	39.9	
**Race**			
White	871	73.3	84.0
Black	134	11.3	12.9
Hawaiian or Pacific Islander	13	1.1	1.3
American Indian	5	0.4	0.5
Asian	4	0.3	0.4
Middle East/North African	1	0.1	0.1
Multiple	9	0.8	0.9
Unknown/not reported	152	12.8	
**Ethnicity**			
Hispanic	292	24.6	27.2
Non-Hispanic	782	65.8	72.8
Unknown/not reported	115	9.7	
**Housing Status**			
Unstably Housed	448	37.7	45.8
Stably Housed	531	44.7	54.2
Unknown/not reported	210	17.7	
**Medical Insurance**			
Medicaid	536	45.1	76.4
Medicare	116	9.8	16.5
Private Insurance	19	1.6	2.7
Other	6	0.5	0.9
No Insurance	25	2.1	3.6
Unknown/not reported	487	41.0	
**Employment Status**			
Unemployed < 1 Year	320	26.9	33.8
Unemployed > 1 Year	437	36.8	46.2
Employed	120	10.1	12.7
Unable to Work	70	5.9	7.4
Unknown/not reported	242	20.4	
**Sex Worker**			
No	817	68.7	95.0
Yes	43	3.6	5.0
Unknown/not reported	329	27.7	
**Highest level of formal education**			
College	98	8.2	12.8
High School/GED	622	52.3	81.3
Less than High School	45	3.8	5.9
Unknown/not reported	424	35.7	
**Primary Language**			
English	1098	92.3	12.8
Other than English	90	7.6	81.3
Unknown/not reported	1	0.1	

Of individuals who disclosed housing status, 54.2% were stably housed and 45.8% were unstably housed. 622 clients (52.3%) completed high school, and 98 (8.2%) of clients were college-educated. Of clients who disclosed employment, 80.0% were unemployed, with 33.8% of the sample unemployed for <1 year, and 46.2% of the sample was unemployed for more than 1 year. Approximately half of clients used state Medicaid for insurance, and most clients (92.3%) spoke English as their primary language.

### Transaction characteristics

**Fig 2:**
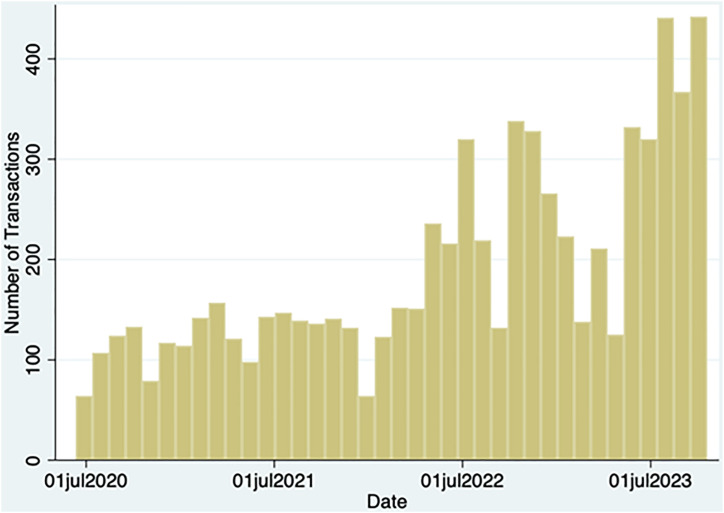
Temporal distribution of transactions.

From the onset of the COVID-19 pandemic (June 2020) to October 2023, there were a total of 7,238 transactions. Transactions increased through the COVID-19 pandemic period (Fig 2). 6,121 (84.6%) of transactions were at the fixed clinic location, followed by 660 (9.12%) home deliveries. During nearly all (7,122; 98.4%) transactions, clients reported using materials for themselves but in 72.1% of transactions, clients reported distribution to groups of 4–6 people, with some individuals distributing materials to groups of up to 15 people. The most frequent injection location reported was home (60.4%), followed by public space (29.8%). All SSP transactions involve, at a minimum, provision of items, and the number of each item distributed is shown in Table 2. During this period, the program dispensed harm reduction supplies, including more than 1,800,000 syringes with a median 133 syringes per month per client. In nearly half of all transactions, teaching was provided on abscess care, safer injection techniques, syringe selection, vein care, and/or wound care.

**Table 2:** Supply distribution and teaching provided.

Supply	Number distributed	Number (%) of transactions
Syringes	1,860,621	
Male condoms	26,690	
Female condoms	54	
Cotton filters	132,437	
Cookers	127,579	
Antibiotic ointment packets	284,637	
Alcohol swabs	357,260	
Bandages	256,415	
Sterile water vials	161,377	
Tourniquets	64,462	
Smoking kits	11,127	
Naloxone kits	970	
Abscess care		3,340 (46.1%)
Safer injection techniques		3,727 (51.5%)
Syringe selection		3,546 (49%)
Vein care		3,187 (44%)
Wound care		3,193 (44.1%)

**Fig 3:**
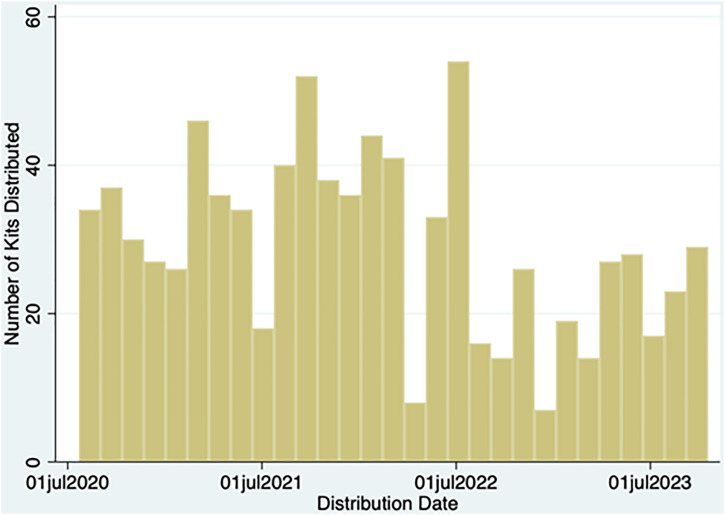
Temporal distribution of naloxone supply.

In addition to the materials noted above, the program provided 970 naloxone kits over 854 distribution events in the observation period. Naloxone distribution peaked in July 2022, decreasing shortly after and into 2023 (Fig 3). There was also documentation of teaching provided on abscess care during 3,340 (46.1%) of transactions, safer techniques (3,727; 51.5%), selecting a syringe (3,546; 49%), vein care (3,187; 44%), and wound care (3,193; 44.1%).

### Testing and evaluation services

Table 3 depicts completion of testing and evaluation services. The subset of clients who had HIV testing and evaluation data available during the observation period (N = 616) were somewhat younger than the larger SSP intake sample, with mean age 38.5 years old (SD 13.1). Testing revealed three new HIV diagnoses, six new cases of syphilis, four new cases of gonorrhea, and no new cases of chlamydia. There were 90 positive screening tests for HCV, though linked data was not available on whether these were newly positive HCV screens or whether HCV RNA was positive. More than half of this subsample completed a comprehensive needs assessment for health benefits, evidence-based risk reduction counseling, behavioral health, and social services, with services provided onsite or referred out accordingly.

**Table 3:** Receipt of testing and evaluation services.

	Total (N = 616)
Mean age, y (SD)	38.5 (13.1)
HIV test done	
Point-of-care	398 (64.6%)
Lab-based	218 (35.4%)
HIV test result	
Negative	613 (99.5%)
Positive	3 (0.5%)
Tested for syphilis	
Yes	217 (35.2%)
Newly identified positive	6 (1.0%)
Negative	206 (33.4%)
Unknown/not reported	5 (0.8%)
No	210 (34.1%)
Missing	189 (30.7%)
Tested for Gonorrhea	
Yes	191 (31.0%)
Positive	4 (0.6%)
Negative	178 (28.9%)
Unknown/not reported	9 (1.5%)
No	236 (38.3%)
Missing	189 (38.3%)
Tested for Chlamydia	
Yes	191 (31.0%)
Positive	0
Negative	182 (29.5%)
Unknown/not reported	9 (1.5%)
No	239 (38.8%)
Missing	186 (30.2%)
Tested for HCV	
Yes	425 (69.0%)
Positive	90 (14.6%)
Negative	330 (53.6%)
Don’t know	5 (0.8%)
No	13 (2.1%)
Missing	178 (28.9%)
Health benefits navigation	
Screened for need	356 (57.8%)
Need determined	41 (6.7%)
Provided/referred	29 (4.7%)
Risk reduction	
Screened for need	358 (58.1%)
Need determined	48 (7.8%)
Provided/referred	32 (5.2%)
Behavioral health	
Screened for need	356 (57.8%)
Need determined	43 (7.0%)
Provided/referred	24 (3.9%)
Social services	
Screened for need	369 (59.9%)
Need determined	63 (10.2%)
Provided/referred	18 (2.9%)

## Discussion

Innovative, low-threshold comprehensive care programs for PWID are urgently needed to meet the challenges presented by the opioid overdose epidemic, including persistently high incidence of fatal overdoses, HIV and HCV [27,28]. Primary care and harm reduction are often siloed [29]. Our evaluation of the New Haven SSP demonstrates a high volume of clients and services, illustrating demand and suggesting it can serve as a model to other harm reduction programs nationally. Many communities have a “syringe gap” between the number of syringes used and the number needed, and current international recommendations for adequate harm reduction coverage is defined as 300 syringes per client per year [30]. Without the limitation of 1-to-1 exchange, our SSP is providing a median 133 syringes per client per month to meet demand.

Existing harm reduction programs should explore if scale-up of clinical services is possible and/or create partnerships with community-based primary care clinics. An initial step could be the expansion of health education activities located at the SSP. One such education-focused intervention provided training to PWID about HIV/ HCV transmission, with an emphasis on injecting technique and HIV/HCV testing and found that participants who received at least one educational session were more likely to undergo HCV testing [16] and emergency department utilization by SSP clients decreased by 20%. Another relatively straightforward activity would be to offer an onsite wound care and abscess clinic. Supervised consumption facilities could be developed in dense areas of substance use to train safe injecting techniques, reduce overdose risk, and support education and referral to treatment. 2021 marked the opening of the nation’s first overdose prevention sites in New York City and in 2022, the Biden administration implemented a National Drug Control Strategy emphasizing high-impact harm reduction interventions. Recent executive orders suggest federal support for harm reduction will not continue, which threatens the sustainability of SSPs nationally [31].

Integration of healthcare and harm reduction services is important to increase access to care but is also cost-effective. While our analysis did not include cost-effectiveness modeling and could not track long-term outcomes, one study found that 50% SSP coverage within the United States (i.e., 50% of PWID have access to and utilize an SSP) would be cost-effective and avert up to 35,000 HIV infections over 20 years [32]. Likewise, community-based distribution of naloxone has been shown to be cost-effective [33,34] and can successfully lower the rate of deaths due to opioid overdose [35,36].

The New Haven SSP navigated some important challenges through the COVID-19 pandemic. The opioid overdose epidemic changed the demographic and geographic characteristics of PWID networks to an increasingly rural and dispersed population [37,38]. Accordingly, our program served a geographically diverse population and some clients traveled >50 miles to receive services. Individuals who do not have close access to SSP must plan their full-day schedules around travel to and from SSP locations, even in a relatively small and urbanized state like Connecticut. The COVID-19 pandemic may have also made travel more difficult, and likely further limited engagement with services. Perhaps the greatest ongoing threat to the New Haven SSP and others is funding support. Sustained funding support from local, state, federal and foundation agencies is crucial to programmatic success. The relative recent dismantling of the Department of Health and Human Services and the Substance Abuse and Mental Health Services Administration, alongside cuts to Medicaid, will most certainly limit the ability of SSPs to provide essential harm reduction services to PWID.

It will be necessary for our program (and others) to develop innovative tools to better communicate scheduling and health information to clients, facilitate use of services (i.e., PrEP and HIV testing), and build trust and engagement with PWID who do not frequently visit the program or rely on others to exchange syringes for them (e.g., secondary exchange). To address this challenge and to better meet the schedules and health needs of clients, the program began providing a home-delivery service to individuals in the greater New Haven area by an outreach worker operating a minivan. A similar home-delivery program in California showed a larger risk reduction of HIV in comparison to a fixed site SSP [39].

Clients are often unwilling to physically remain at an SSP for long, stemming from fear of law enforcement, desire not to be seen by peers (i.e., stigma, shame), and experiencing withdrawal symptoms. Certain characteristics, such as secondary distribution to others, can be risky to report. As a result, the program has challenges engaging clients with its full range of services (i.e., HIV rapid testing, PrEP initiation, health education) and scaling up its home-delivery program. In response, services are now promoted through a variety of platforms, including flyers, two websites, and a profile on Grindr (a geosocial dating app oriented to gay and bisexual men). In addition, staff frequently attend health fairs and provide workshops to local schools to better engage with members of the community who may not directly receive syringe services from the program. One innovative strategy to address these challenges would be the development of a “harm reduction” app that could be distributed free-of-charge. The app could allow clients to order safe injecting equipment needed either as “pick up” or as a delivery. For those clients who want more medical services, the app could also allow clients to “opt in” and be linked to a clinic’s (i.e. MMC’s) electronic health record using HIPAA compliant encryption. This would allow for rapid HIV, HCV, hepatitis B (HBV), and sexually transmitted infection (STI) testing and facilitate necessary laboratory tests needed for PrEP initiation and HCV treatment. The app could also allow the program to distribute educational materials (e.g., videos, brief messages, etc.) to reach a wider audience.

## Conclusions

SSPs are a critical component of HIV, HCV, and overdose prevention among PWID because they offer non-judgmental, free, and accessible testing and care services to PWID who may not contact other medical services. Amid a volatile opioid epidemic with increasing risk for HIV and HCV, SSPs must provide a larger array of health services than previously offered by many programs. The New Haven SSP is a fully integrated harm reduction and healthcare service program for PWID and may serve as a model for other SSPs in the United States navigating similar challenges.
